# Supplementary material for: Content-rich biological network constructed by mining PubMed abstracts
Source: BMC Bioinformatics. 2004 Oct 8;5:147. doi: 10.1186/1471-2105-5-147 (PMC528731; doi:10.1186/1471-2105-5-147)
Supplement: Additional File 2 — The original results of the above study (non-essential files are deleted to keep the file size under the limit set by BMC bioinformatics). [file 1471-2105-5-147-S2.bz2 › chilibotAdditionalFile2/dip05/472ID9195914E10538/html/IL8RA_CXCL1.html]

 


 **IL8RA** and **CXCL1** 
  
Found 47 abstracts in PubMed, retrieved 05.  
 

 What does Google say? 
 PDF only 
| .edu only 

---

- Biochem Pharmacol, 2003   **Characterization of the molecular interactions of interleukin 8 CXCL8 , growth related oncogen alpha **CXCL1** and a non peptide antagonist SB 225002 with the human CXCR2.** .
  Neutrophil recruitment to inflammatory sites is mediated by two related receptors CXC chemokine receptors 1 CXCR1 **[IL8RA]** and 2 CXCR2 .
  Both receptors share two ligands, interleukin 8 CXCL8 and GCP 2 CXCL6 .
  . . . several chemokines, including growth related oncogen alpha **CXCL1** and a non peptide antagonist SB 225002 are specific for CXCR2.
  The objective of this study was to map the different amino acids involved in the binding and activation inhibition of human CXCR2.
  This was performed by exchanging non conserved amino acids of CXCR2 with their counterparts in CXCR1 **[IL8RA]**.
  The mutants generated showed that a for CXCL8 binding, the N terminus of CXCR1 **[IL8RA]** and the second extra cellular loop of CXCR2 are determinant, the N terminus of CXCR2 is not sufficient and the transmembrane domain seven is probably involved.
  b for **CXCL1**, the N terminus of CXCR2 is necessary but not sufficient for binding.
  The activation study indicated that amino acids critical for activation are not necessarily involved in binding process.
  Finally, the mechanism of binding of a non peptide antagonist on CXCR2 was investigated it occurred through epitopes a which were disseminated within the receptor, b which differed according to the use of CXCL8 or **CXCL1** as a competitor and c which did not necessarily overlap with agonist binding sites.
  We also showed that inhibition of binding and inhibition of activation involved different amino acids.

  - Int Immunol, 2002   **Systemic chemokine and chemokine receptor responses are divergent in allergic versus non allergic humans.** .
    T h 1 and T h 2 polarized human T cell clones display distinct patterns of chemokine receptor expression and selective chemokine responsiveness in vitro.
    We hypothesized that natural exposure to environmental grass pollen would induce differential systemic chemokine and chemokine receptor expression patterns in individuals with allergic rhinitis compared to healthy controls with type 2 and type 1 dominated responses to allergen respectively.
    To this end, we compared chemokine receptor expression on peripheral blood T cells directly ex vivo and plasma chemokine levels between these two groups of study participants prior to and during the grass pollen season.
    T h 1 associated CXC chemokine receptor CXCR **[IL8RA]** 3 was strongly expressed on >50% CD4 CD45RO cells of all subjects.
    When examined longitudinally, CXCR3 expression increased over the grass pollen season P <.
    0.0001 , solely in non allergic subjects.
    In contrast, for both allergic and non allergic subjects, CC chemokine receptor CCR 5 T h 1 associated and CCR3 T h 2 associated were weakly expressed on <10% of CD4 CD45RO cells both prior to and during the grass pollen season.
    Type 1 chemokines CXC chemokine ligand CXCL **[CXCL1]** 9 and CXCL10 monokine induced by IFN gamma and IFN gamma inducible protein of 10 kDa CXCR3 ligands , and type 2 chemokines CC chemokine ligand CCL 11 eotaxin CCR3 ligand , CCL17 thymus and activation regulated chemokine CCR4 ligand and CCL22 monocyte derived chemokine CCR4 ligand were readily detectable in the plasma of most participants.
    Systemic CXCL9 levels decreased from pre to grass pollen season in allergics P <.
    0.05 .
    . . . CCL17 decreased in non allergics P <.
    0.05 over the same period.
    Taken together, these longitudinal data suggest a systemic shift to more intensely type 1 dominated responses in non allergic individuals and, conversely, to more type 2 dominated responses in allergic individuals upon natural re exposure to grass pollen.

    - Vet Immunol Immunopathol, 2002   **CXCL8 3 73 K11R G31P antagonizes the neutrophil chemoattractants present in pasteurellosis and mastitis lesions and abrogates neutrophil influx into intradermal endotoxin challenge sites in vivo.** .
      The ELR CXC chemokines are critical for protective neutrophil responses to most bacterial infections, but nevertheless can contribute importantly to the pathogenic effects of many inflammatory responses.
      We recently engineered a series of high affinity CXCL8 IL 8 antagonists, one of which, CXCL8 3 73 K11R G31P, binds very strongly to neutrophils via the CXCR1 **[IL8RA]** and CXCR2.
      Herein we show in competitive 125I ligand binding assays that bovine CXCL8 3 73 K11R G31P has an affinity for neutrophils that is 2 3 orders of magnitude higher than that of CXCL8 IL 8.
      Furthermore, when used at approximately 0.5 nM, CXCL8 3 73 K11R G31P inhibited by 50% the chemotactic responses of neutrophils to 129 nM CXCL8 IL 8, but it also blocked chemotactic responses to the alternate ELR CXC chemokines **CXCL1** GRO **[CXCL1]** alpha and CXCL5 ENA 78.
      Furthermore, CXCL8 3 73 K11R G31P could inhibit by 93 97% the spectrum of neutrophil chemotactic activities present within wash fluids from clinical bacterial pneumonia or experimental endotoxin induced mastitis lesions.
      Finally, intramuscular or subcutaneous application of CXCL8 3 73 K11R G31P 75 micro g kg reduced by up to 97% neutrophil infiltration into intradermal endotoxin challenge sites in cattle, and prevented their circulating neutrophils from responding to CXCL8 IL 8 or ENA 78 in vitro.
      This data thus encourages further investigation of the potential impact of this novel antagonist on ELR CXC chemokine driven inflammatory disorders.

      - Fish Shellfish Immunol, 2002   **Molecular cloning and genomic structure of an interleukin 8 receptor like gene from homozygous clones of rainbow trout Oncorhynchus mykiss .** .
        Chemokines are small proteins 70 100 amino acids which play an important role in recruitment and activation of leucocytes to migrate to the site of inflammation.
        Based on the position of the first two conserved cysteines, chemokines are classified into four subfamilies C, CC, CXC and CX3C.
        To date, many members of CC and CXC have been found and studied extensively 1 .
        Chemokines exert effects on their target cell via chemokine receptors, which are G protein coupled receptors containing seven transmembrane domains with an extracellular N terminus and an intracellular C terminus 2 .
        Interleukin 8 IL 8 belongs to the CXC chemokine subfamily.
        It can activate and attract migratory neutrophils to an inflammation site.
        Two IL 8 receptors, CXCR1 **[IL8RA]** and CXCR2, have been identified in mammals 3 6 .
        both of these receptors have high affinity for IL 8 and are expressed on the neutrophil.
        CXCR1 **[IL8RA]** just binds IL 8.
        CXCR2 binds IL 8 and other structurally related chemokines such as growth related oncogene GRO **[CXCL1]** a, GRObeta, GROgamma, neutrophil activating peptide 2 NAP 2 and epithelial cell derived neutrophil activating peptide 78 ENA 78 .
        Several studies on fish chemokine receptors have been reported 9 11 .
        Thus far IL 8 and CXCR1 **[IL8RA]** and CXCR2 proteins from rainbow trout have not been reported however, the sequence of a rainbow trout IL 8 has been noted GenBank Accession No.
        AJ279069 12 .
        Cloning of the IL 8 receptor is important to study the function of IL 8 CXCR1 **[IL8RA]** and CXCR2 in inflammation and signal transduction in fish.
        This paper reports the molecular cloning and genomic structure of an IL 8 receptor like gene from four homozygous clones of rainbow trout Oregon State University OSU , Hot Creek HC , Arlee AR and Swanson SW .

        - J Immunol, 2002   **Platelet derived chemokines CXC chemokine ligand CXCL **[CXCL1]** 7, connective tissue activating peptide III, and CXCL4 differentially affect and cross regulate neutrophil adhesion and transendothelial migration.** .
          In this study, we have examined the major platelet derived CXC chemokines connective tissue activating peptide III CTAP III , its truncation product neutrophil activating peptide 2 CXC chemokine ligand 7 CXCL7 , as well as the structurally related platelet factor 4 CXCL4 for their impact on neutrophil adhesion to and transmigration through unstimulated vascular endothelium.
          Using monolayers of cultured HUVEC, we found all three chemokines to promote neutrophil adhesion, while only CXCL7 induced transmigration.
          Induction of cell adhesion following exposure to CTAP III, a molecule to date described to lack neutrophil stimulating capacity, depended on proteolytical conversion of the inactive chemokine into CXCL7 by neutrophils.
          This was evident from experiments in which inhibition of the CTAP III processing protease and simultaneous blockade of the CXCL7 high affinity receptor CXCR **[IL8RA]** 2 led to complete abrogation of CTAP III mediated neutrophil adhesion.
          CXCL4 at substimulatory dosages modulated CTAP III as well as CXCL7 induced adhesion.
          Although cell adhesion following exposure to CTAP III was drastically reduced, CXCL7 mediated adhesion underwent significant enhancement.
          Transendothelial migration of neutrophils in response to CXCL7 or IL 8 CXCL8 was subject to modulation by CTAP III, but not CXCL4, as seen by drastic desensitization of the migratory response of neutrophils pre exposed to CTAP III, which was paralleled by selective down modulation of CXCR **[IL8RA]** 2.
          Altogether our results demonstrate that there exist multiple interactions between platelet derived chemokines in the regulation of neutrophil adhesion and transendothelial migration.
